# WinRoots: A High-Throughput Cultivation and Phenotyping System for Plant Phenomics Studies Under Soil Stress

**DOI:** 10.3389/fpls.2021.794020

**Published:** 2022-01-28

**Authors:** Yangyang Zhang, Wenjing Zhang, Qicong Cao, Xiaojian Zheng, Jingting Yang, Tong Xue, Wenhao Sun, Xinrui Du, Lili Wang, Jing Wang, Fengying Zhao, Fengning Xiang, Shuo Li

**Affiliations:** ^1^The Key Laboratory of Plant Development and Environmental Adaptation Biology, Ministry of Education, School of Life Sciences, Shandong University, Qingdao, China; ^2^Weifang Academy of Agriculture Sciences, Weifang, China

**Keywords:** high-throughput plant cultivation, root phenotyping, whole-seedling phenotyping, plant stress phenotyping, abiotic stress

## Abstract

Soil stress, such as salinity, is a primary cause of global crop yield reduction. Existing crop phenotyping platforms cannot fully meet the specific needs of phenomics studies of plant response to soil stress in terms of throughput, environmental controllability, or root phenotypic acquisition. Here, we report the WinRoots, a low-cost and high-throughput plant soil cultivation and phenotyping system that can provide uniform, controlled soil stress conditions and accurately quantify the whole-plant phenome, including roots. Using soybean seedlings exposed to salt stress as an example, we demonstrate the uniformity and controllability of the soil environment in this system. A high-throughput multiple-phenotypic assay among 178 soybean cultivars reveals that the cotyledon character can serve as a non-destructive indicator of the whole-seedling salt tolerance. Our results demonstrate that WinRoots is an effective tool for high-throughput plant cultivation and soil stress phenomics studies.

## Introduction

Stress due to soil composition, such as salinity, heavy metals, and nutrient deficiency, is a primary cause for crop yield reduction (Miransari, 2010; Mcdonald et al., 2017; Zorb et al., 2019; Siao et al., 2020). This soil-related stress is first sensed by roots and affects their functions, leading to changes in plant physiology and biochemical reactions, ultimately influencing plant growth and yield (Li et al., 2018; Khan et al., 2019). The identification of genes linked to soil stress responses and assessment of how they contribute to combating stress drive molecular design breeding to develop stress-resistant and high-yielding crops, which have become vibrant topics for research. Crop soil stress tolerance is a complex trait that involves the regulation of a variety of genetic and non-genetic factors such as plant morphology, metabolism, and gene regulatory networks (Cui et al., 2015; Yang and Guo, 2018). However, fully revealing the underlying molecular genetic networks and effectively harnessing them to guide molecular breeding has been extremely challenging. In recent years, the rapid development of high-throughput omics techniques, including phenomics platforms, has opened new avenues for the study of complex genetic traits in plants. Through integrated analyses such as phenotype-genotype association studies, it is now possible to comprehensively describe the plant genetic regulatory networks that respond to soil stress and apply this new knowledge to breed stress-resistant crops (Edwards and Batley, 2004; Grosskinsky et al., 2015; Jie, 2015; Al-Tamimi et al., 2016; Liu et al., 2016; Bevan et al., 2017; Muthamilarasan et al., 2019). At present, while next-generation sequencing technology has become more popular and accessible, high-throughput plant phenotyping has become the main bottleneck for large-scale functional analyses (Tardieu et al., 2017; Bolger et al., 2019; Wu et al., 2020).

Traditional crop phenotyping is generally carried out in the field, which is time-consuming and labor-intensive, suffers from low throughput, and can routinely be affected by natural environmental factors beyond the control of the researcher. Under such conditions, it is hard to obtain highly accurate phenotypic data to meet the requirements of phenomics studies. In past decades, several HTP (high-throughput phenotyping) platforms have been developed for use in the field or under controlled conditions. These platforms rely on automated transmission and control technologies. Various types of computer vision sensors, non-invasive imaging, and image analysis technologies can be used to perform phenotypic analysis on dozens, hundreds, or even thousands of plants over a short period of time (Furbank and Tester, 2011; Junker et al., 2014; Ghanem et al., 2015; Araus et al., 2018). The application of these tools to high-throughput phenotyping in controlled-environment facilities has the potential to improve accuracy and reduce the need for replication (Furbank and Tester, 2011). For example, the indoor automated robot phenotyping platform Phenoscope can accommodate the simultaneous cultivation of 735 Arabidopsis (*Arabidopsis thaliana*) plants and is equipped with a zenithal imaging system to monitor rosette size and expansion rate during the vegetative stage (Tisne et al., 2013). Similarly, the high-throughput rice (*Oryza sativa*) phenotyping identification facility (HRPF), which combines color imaging, X-ray computed tomography (CT), automatic controls, and image analysis functions, can extract at least 15 agronomic features from 533 rice cultivars grown in the greenhouse (Yang et al., 2014). PlantScreen™ (PSI, Czech Republic), a large-scale plant phenotyping imaging analysis platform equipped with a conveyor belt, was used to evaluate the changes in growth, morphology, and photosynthetic performance of nine Arabidopsis accessions exposed to salt stress (Awlia et al., 2016). These high-throughput phenotyping platforms can quickly obtain precise phenotypic values from potted plants, but their operation and maintenance costs are extremely high. In addition, research aimed at crop phenotyping usually focuses on the aboveground parts of plants while the acquisition of data from root morphology is much more limited (Chen et al., 2007; de Dorlodot et al., 2007; Meister et al., 2014; Wu et al., 2015; Crowell et al., 2016; Choudhury et al., 2019; Li M. et al., 2020; Nagel et al., 2020).

Roots, however, are the major route for water and nutrient uptake in plants, and they may also serve as storage organs for carbohydrates, as well as the direct sensing organ of soil stress (Zhu et al., 2011). Root morphology and root physiological functions change under soil stress (Ho et al., 2004; Zhu et al., 2005; Reynolds et al., 2007; York et al., 2013; Al-Tamimi et al., 2016). Therefore, phenotyping roots is an integral part of studying the plant phenome under soil stress conditions. To date, several root phenotyping technologies have been developed under controlled conditions. These can be divided into two primary categories. One subgroup relies on liquid growth medium (Ingram et al., 2012; Clark et al., 2013; Wu et al., 2018), germination paper (Le Marie et al., 2014; Atkinson et al., 2015; in ‘t Zandt et al., 2015; Gioia et al., 2017), solid growth medium (Busch et al., 2012), and other non-soil matrix growth systems. The second set of methods uses soil (Manschadi et al., 2006; Nagel et al., 2012). Non-soil matrix systems can easily achieve the direct quantification of root morphology and allow the non-invasive imaging analysis of numerous roots in a short time. However, non-soil technology cannot completely mimic the effects of soil on root development (Bengough et al., 2004; Hargreaves et al., 2009) and may even introduce unwanted variables such as hypoxia and bacterial growth (Zhu et al., 2011). To solve these problems, several groups have developed root phenotyping methods specifically designed for soil-grown plants. Although these methods achieved *in situ* observation and quantitative analysis of the root system by imaging transparent soil-filled columns or rhizotrons, their associated throughput was limited to fewer than 100 plants (Manschadi et al., 2006, 2008; Nagel et al., 2012). Advances in X-ray CT and magnetic resonance imaging (MRI) now allow non-invasive three-dimensional (3D) imaging of crop roots grown in opaque substrates that can quantify root system characteristics (Perret et al., 2007; Tracy et al., 2010; Clark et al., 2011; Mairhofer et al., 2013; Metzner et al., 2015; Pfeifer et al., 2015; Rogers et al., 2016; van Dusschoten et al., 2016). However, the high cost of CT and MRI equipment and the long scanning time needed for individual samples limit the application of these approaches in high-throughput root phenotyping platforms (Gregory et al., 2009; Zhu et al., 2011). Therefore, developing a high-throughput soil cultivation and whole-plant phenotyping system suitable for crop soil stress research remains a largely unfinished task.

Here, we describe the recently developed WinRoots platform, a high-throughput plant cultivation and phenotyping system for plants grown in a soil matrix. WinRoots allows the dynamic monitoring and acquisition of whole-plant phenotypes from plants grown in high throughput in uniform and controllable soil stress conditions. It represents an economical and flexible system with significant space savings compared to other methods. We phenotyped soybean (*Glycine max*) plants exposed to salt stress as a proof of concept and demonstrated the uniformity and controllability of soil stress conditions and the high throughput of the WinRoots system. We developed optimized salt stress conditions and phenotypic indices suitable for high-throughput phenotyping of salt tolerance in soybean. In addition, a high-throughput multiple-phenotypic analysis indicates that the cotyledon character can serve as a useful non-destructive indicator of the whole-seedling salt tolerance in soybean. This new system will be a powerful tool for studying the complex traits of crops in response to soil stress.

## Materials and Equipments

### Structure of WinRoots System

**FIGURE 1:**
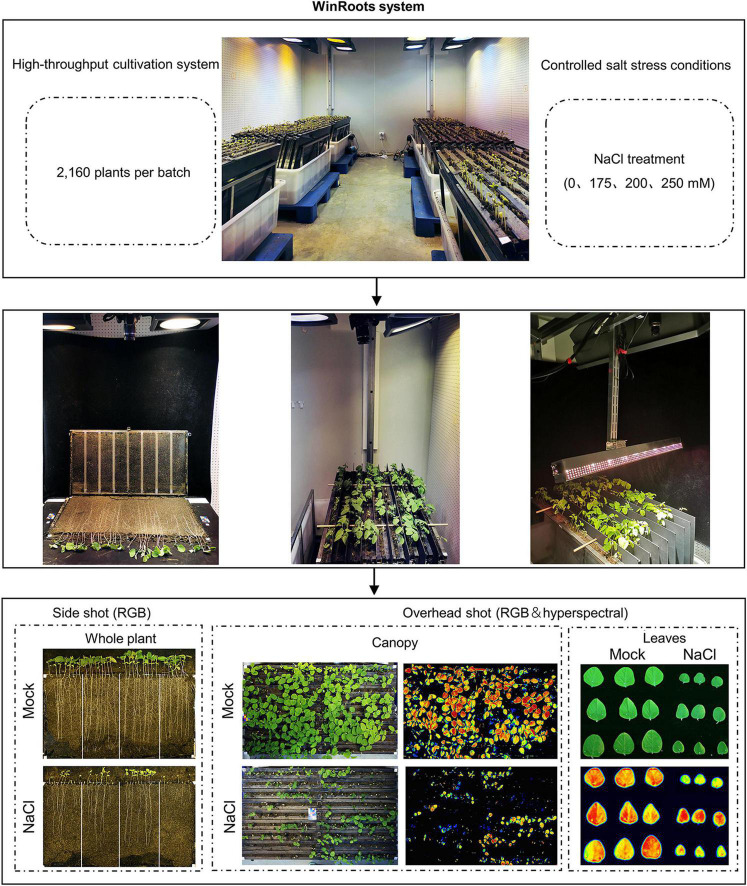
The WinRoots system: a soil-based, high-throughput plant culture platform for phenotyping roots and whole plants. Schematic representation of the usage of the system.

**FIGURE 2:**
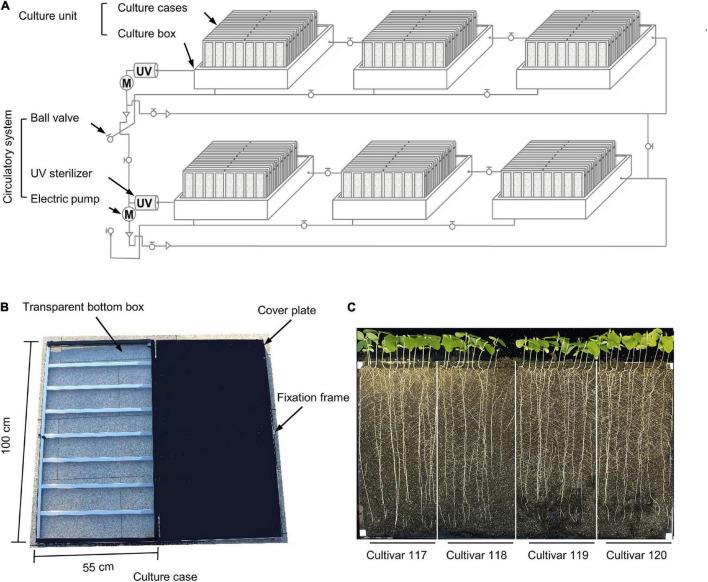
Individual components of the WinRoots system. **(A)** Schematic diagram of all components of the WinRoots system. **(B)** Structure of a single culture case. **(C)** Representative images of whole seedlings from four soybean cultivars grown in the same culture case for 10 days. Bar, 5 cm.

As shown in Figure 1, the WinRoots system consists of two or more independent circulatory systems and several plant culture units. The circulatory system is composed of electric pumps, an ultraviolet (UV) sterilizer, and pipes equipped with ball valves (Figure 2A). When turned on, the electric pumps promote the circulation of the liquid in the system when in use. It also help discharge the liquid out of the system in between experiments. A UV sterilizer irradiates the liquid flowing through the water pipes to inhibit the growth of algae, bacteria, and other microorganisms, thus avoiding contamination of the liquid environment that would otherwise affect root growth. The pipes are fitted with ball valves and connect the culture boxes; opening or closing the ball valve can form a shared and homogeneous liquid environment between boxes or an independent liquid environment for each culture box (Figure 2A). In this experiment, the circulatory system worked 1 h per day every 12 h to maintain the uniform of the electrical conductivity in the soil matrix.

Each culture unit comprises several culture cases stacked obliquely in a culture box. The inner wall of each culture box is 106 cm (length) × 79 cm (width) × 32 cm (height). Each box can accommodate nine culture cases stacked obliquely. Each culture case consists of a 100 cm × 55 cm × 2 cm box with a transparent bottom and a cover plate of the same size (Figure 2B). There are 120 vent holes with a 1-mm diameter, evenly distributed over the surface of the cover plate. To maintain a constant distance of 2 cm between two consecutive plates inside the culture case, a metal fixation frame surrounds each culture case (Figure 2B).

The transparent bottom box is filled with soil matrix and is then assembled by placing the cover plate over the transparent box and fixed with an openable clip frame to provide the soil environment for plant growth. The culture box serves as a container for water or culture solution, from which the soil in the culture cases can draw water through capillary action.

### Plant Material

Experiments were conducted on plants from 178 soybean cultivars harvested in 2019. The seeds were surface sterilized in 75% ethanol for 3 min, rinsed in sterile water three times, and then placed in a moist seed germination box to germinate at 25°C in the dark. After 48 h, seedlings with visible radicle elongation of about 1 cm were selected and transferred to culture cases and planted at a depth of 2 cm. The matrix in the culture cases was composed of nutritive soil and vermiculite at a ratio of 3:1, mixed with a fixed volume of water or NaCl solution at a specific concentration. The WinRoots system was placed in the climate chamber. Environment parameter settings were as follows: light/dark = 16 h/8 h, temperature 25°C, and humidity 75%. Though the system can support the soybean seedlings grow normally to stage V_2_, in this experiment, the seedlings grow to stage V_1_ (∼ 10 days after planting).

## Methods

### Experimental Design

The version of the WinRoot system presented here is equipped with six culture units, each of which contains nine culture cases that allow visual inspection of root growth. Each culture case can accommodate 40 soybean seedlings for normal growth (with spacing between neighboring seedlings of about 2.5 cm), and each experiment can accommodate up 2,160 soybean seedlings for simultaneous growth (Figures 1, 2A,C and Supplementary Figure 1). By adjusting the number or size of culture units and culture cases, the throughput of the WinRoots system can be adjusted at the user’s discretion to suit their space and plant material requirements. When setting up an experiment, the emerging radicle of a soybean seed should be placed close to one side of the transparent bottom box. The planting design for uniformity assay and salt stress condition determination was shown in Supplementary Figure 1.

The culture case should be positioned obliquely so that the transparent side forms a 66° angle with the ground and remains in this position during the entire course of the experiment. Root geotropism will promote growth along the transparent side, thus allowing the unobstructed observation of plant roots.

Before the start of the experiment, it is critical to ensure that the liquid level in all culture boxes reaches 10 cm above the bottom of the culture cases; six culture boxes are placed symmetrically in pairs and arranged on the floor support of the climate chamber at equal intervals. The culture boxes located on the same side are connected to each other through the water inlet and outlet pipes with ball valves. Opening ball valves allow liquid circulation across adjacent culture boxes and ensure that the soil in all visible culture boxes receives the same uniform and stable external liquid environment.

Each culture case in the culture box was divided into four site intervals. Each site comprised 10 seedlings from one cultivar. The primary roots of soybean seedlings cultured in the WinRoots system grew straight down along the transparent wall. After 10 days of growth under mock conditions, the root tips reached the bottom of the culture case. Red-green-blue (RGB) images or hyperspectral data can be collected for whole seedlings from the transparent side of the case or for the seedling canopy from the top of the culture units during cultivation, respectively (Figures 1, 2C), and then analyzed to extract phenotypic trait values.

### Conductivity Tests

A portable handheld soil conductivity meter (0–10,000 μs/cm) was used to measure soil conductivity. Soil conductivity was measured about 5 cm below the soil surface at four sites 25 cm apart in each culture case, at 1 day before planting and 10 days after planting.

### Red-Green-Blue and Hyperspectral Data Acquisition

A Canon EOS 700D digital camera and a Resonon PIKA L hyperspectral imager were used to collect RGB (in raw format) and hyperspectral images, respectively. The camera was positioned from a slidable horizontal rail 1.5 m above the plant material. The images of soybean canopy (overhead shot) and of the whole seedlings (side shot) were collected every day. On the 9th day of cultivation, images were taken of detached leaves with three replicates for each cultivar.

### Phenotype Measurement

ImageJ version 1.53a^1^ software was used to measure primary root length (PRL) and leaf area from RGB images (in tiff format). After imaging whole seedlings on the 9th day, the shoot part and cotyledons of each seedling were harvested separately and their fresh weight determined. The samples were then dried at 100°C for 3 days and their dry weight measured. We calculated the water content and water weight per plant based on the formula WC = (average fresh weight – average dry weight)/average fresh weight and WW = average fresh weight – average dry weight. The relative index = the index of seedlings grown under salt stress/the index of seedlings grown in mock condition.

### Data Analysis

Microsoft Excel 2016 with XLSAT plug-in (Ver. 2019.2.2) was used for data analyses. The Student’s *t*-test was used to check whether two sets of data differ significantly. For correlation analysis, the Spearman correlation test was performed on the phenotypic data.

For agglomerative hierarchical clustering, the phenotypic indices was standardized, the similarity was evaluated with Kendall correlation coefficient, and the unweighted pair-group average agglomeration method was used.

## Results

### Uniform Soil Cultivation Environment in WinRoots System

Most high-throughput crop phenotyping efforts are performed in pots (one plant per pot) or on germination paper (several plants per sheet of paper) (Adu et al., 2014; van Dusschoten et al., 2016), making it difficult to ensure that the “micro-environment” of these materials is consistent throughout the entire system. To solve this problem, we established WinRoots (Figures 1, 2A), a high-throughput plant cultivation and phenotyping system that is based on a soil matrix and offers root visibility.

**FIGURE 3:**
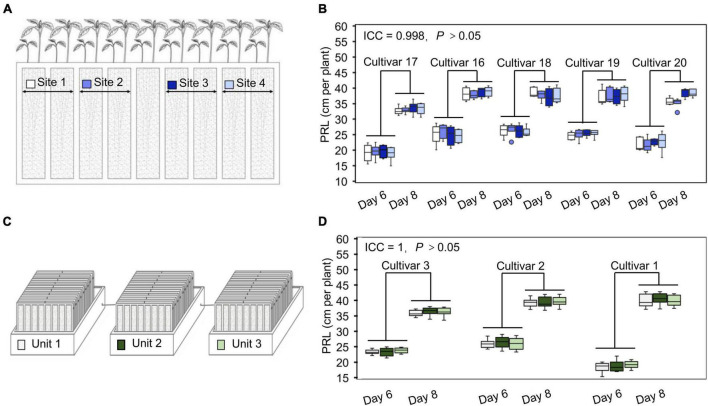
The WinRoots system provides high uniformity of conditions between culture sites. **(A)** The different positions in each culture case are assigned a site number from 1 to 4, with each site supporting the growth of at least 10 soybean seedlings. **(B)** Primary root length (PRL) of soybean seedlings growing in different sites within the same culture case for 6 or 8 days. **(C)** Three culture units under normal conditions are assigned a number from 1 to 3. Each unit contains nine culture cases. **(D)** PRL of soybean seedlings growing in different units under normal conditions for 6 or 8 days. ICC, intraclass correlation coefficient; *P*, *P*-value.

Changes in environmental factors can drastically affect measured plant phenotypes. To partially account for these effects and accurately identify phenotypic differences between cultivars, one strategy is to include a large number of replicates to dilute any variation caused by an uneven environment. However, this will also increase the cost of phenotyping. An alternative way of obtaining accurate phenotypic data at an affordable cost is the establishment of highly controlled and stable cultivation conditions with limited repetitions. To evaluate the uniformity of the soil cultivation environment in the WinRoots system, we used soybean PRL as an indicator to analyze the potential for position effects at different sites within one culture case and across different culture boxes. For this test, the WinRoots system was placed inside a climate-controlled growth chamber to minimize variation in temperature and humidity and used to collect data for 520 seedlings from 7 soybean cultivars. We determined PRL for four soybean cultivars placed at four different sites after 6 and 8 days of growth to test the position effect within culture cases, each site containing 10 replicate seedlings. Our results showed that root length was very consistent for each cultivar across the four sites tested, as the intraclass correlation coefficient (ICC) was 0.998, with an associated *P*-value of > 0.05 (Figures 3A,B and Supplementary Figure 1). Similarly, we evaluated PRL for three soybean cultivars across three different culture boxes to assess the possible contribution of the culture boxes to variation. Again, root length displayed high consistency, with an ICC of 1 and *P* > 0.05 (Figures 3C,D). These results indicate that the WinRoots system can provide a uniform soil culture environment for soybean seedlings and that the phenotypic data obtained under these conditions are accurate and reliable.

### Precise Control of Soil Salt Stress Conditions

**FIGURE 4:**
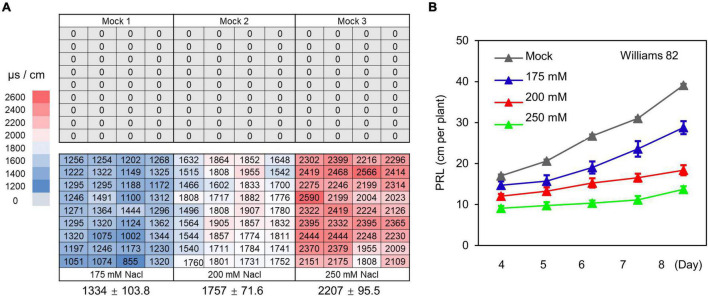
The WinRoots system provides controlled salt stress conditions. **(A)** Electrical conductivity of surface soil in the WinRoots system (10 days after planting). Each number indicates the electrical conductivity of a sample site within a culture case. The average electrical conductivity (*n* = 36) and the standard deviation (SD) are shown below the image. **(B)** PRL of soybean cultivar Williams 82 under mock and salt stress conditions. Data are shown as mean value ± standard error, *n* = 15.

Soil stress conditions in the field are often uneven, which constitutes a major limiting factor for obtaining accurate phenotypes related to soil stress in field-grown crops. To test whether the WinRoots system can provide soil salinity conditions that are precisely controlled and highly homogeneous, we evaluated soil conductivity at three different salt stress levels. We used an equal volume of water (mock) or NaCl solution (175, 200, and 250 mM) to soak the nutritive soil-vermiculite mixture and then fill the culture cases, and then placed the culture cases in different culture boxes (Supplementary Figure 1). Germinating soybean seeds were sown after the circulatory system had been allowed to run for 1 day. We planted seedlings at the same positions within each unit for mock and salt stress conditions (Supplementary Figure 1). Soil conductivity was measured for a total of 216 sites sampled, 1 day before and 10 days after planting (Figure 4A and Supplementary Figure 2). Although we observed significant differences in soil conductivity between different conditions, as expected, the variation in conductivity at different positions for a given condition was very small (Figure 4A and Supplementary Figure 2). This indicates that soil conditions in the WinRoots system are uniform and controllable when the system is used to impose high salinity stress.

Next, we determined PRL for the soybean cultivar Williams 82 exposed to the above salt stress conditions for 4–8 days. We observed an inhibition of primary root growth by all salt stress conditions that was commensurate with the salt concentration. The difference in PRL between seedlings exposed to various salt concentrations increased gradually over time (Figure 4B). Together, these results indicate that the WinRoots system can evoke uniform and controlled salt stress when seedlings are grown in a soil matrix and allows the collection of precise seedling phenotypes.

### Determination the Optimal Salt Stress Condition in Soybean Salt Tolerance Screen

In a larger-scale experiment, we used root length, fresh weight, and water content of 11 soybean cultivars under three salt stress conditions in the WinRoots system to define the appropriate conditions to phenotype salt stress tolerance.

**FIGURE 5:**
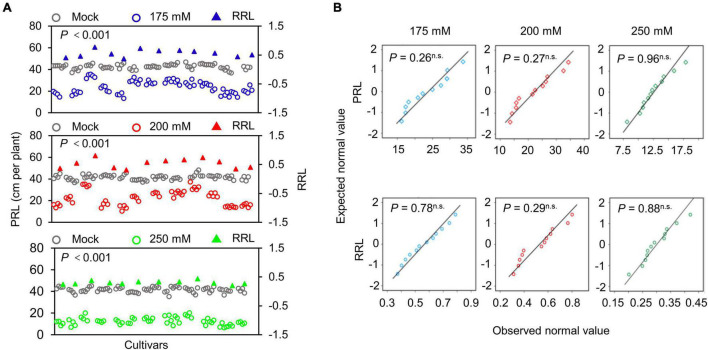
Determination of primary root length. **(A)** Scatter distribution map of PRL (circles) and relative primary root length (RRL, triangles) of soybean seedlings. Gray, blue, red, and green represent the soybean seedlings grown under mock treatment or NaCl treatment of 175, 200, or 250 mM, respectively. Each circle represents the PRL of an individual seedling. Each triangle represents the value of the RPL of a soybean cultivar. Data were collected 10 days after planting. A paired Student’s *t*-test was conducted between mock and NaCl-treated seedlings to test for significance. **(B)** Quantile-quantile plots of PRL and RRL. The normality of the data was tested using the Shapiro-Wilk test. *P*, *P*-value; n.s., not significant. The planting design is shown in Supplementary Figure 1.

The structure of the root system at the seedling stage largely reflects the growth of newly emerged seedlings experiencing stress to support seedling growth and development (de Dorlodot et al., 2007; Meister et al., 2014; Wu et al., 2015). To evaluate the differences in soybean seedlings under different salinity levels, we measured the PRL of 720 individual seedlings from 11 soybean cultivars either grown under normal conditions (mock treatment) or exposed to 175, 200, or 250 mM NaCl for 10 days. To evaluate the salt stress sensitivity of the seedlings, we use the relative root length (RRL) as an index. Soybean PRL was significantly lower in all three salt stress conditions tested here than in the mock treatment (Figure 5A, *P* < 0.001). In addition, PRL showed greater variation at the salt concentrations of 175 and 200 mM NaCl, suggesting that these two conditions may be used as sensitized stress conditions to screen root-related salt stress phenotypes with the WinRoots system. Importantly, all recorded data followed a normal distribution in all conditions (Figure 5B, *P* > 0.05).

We also measured the fresh weight (FW), the water content (WC) and calculated their relative indices (relative fresh weight, RFW; relative water content, RWC) of the aboveground parts of soybean seedlings. As with PRL, all salt stress conditions significantly reduced FW and WC relative to mock conditions, and at 175 mM NaCl condition the indices showed higher variation (Supplementary Figures 3A, 4A, *P* < 0.001). All indices followed a normal distribution (Supplementary Figures 3B, 4B, *P* > 0.05).

Based on the above observations, we determined that 175 mM NaCl was a suitable salt stress condition to quantify salt tolerance and sensitivity in a soybean population by the WinRoots system.

### High-Throughput Multiple-Phenotypic Assay Reveals Cotyledon Character as a Useful Indicator for Salt Tolerance Estimation in Soybean

To access the soybean salt tolerance on a multiple-phenotype scale, we turned next to the high-throughput analysis of 146 soybean cultivars and recorded whole-seedling phenotypes such as the side (on day 10th) and leaf images (on day 9th) after planting (Supplementary Figure 5). We measured the PRL and leaf area (LA) of plants in both mock and NaCl treatment. In addition, we measured the FW and DW of the cotyledons and shoots for the same cultivar. Based on these data, we obtained 18 indices to access the state of soybean seedlings in mock condition and under salt stress. The indices included the PRL, LA, FW of shoots or cotyledons, water weight (WW) of shoots or cotyledons, and their corresponding relative indices (salt stress/mock). All these indices were salt stress affected (Supplementary Figure 6).

**FIGURE 6:**
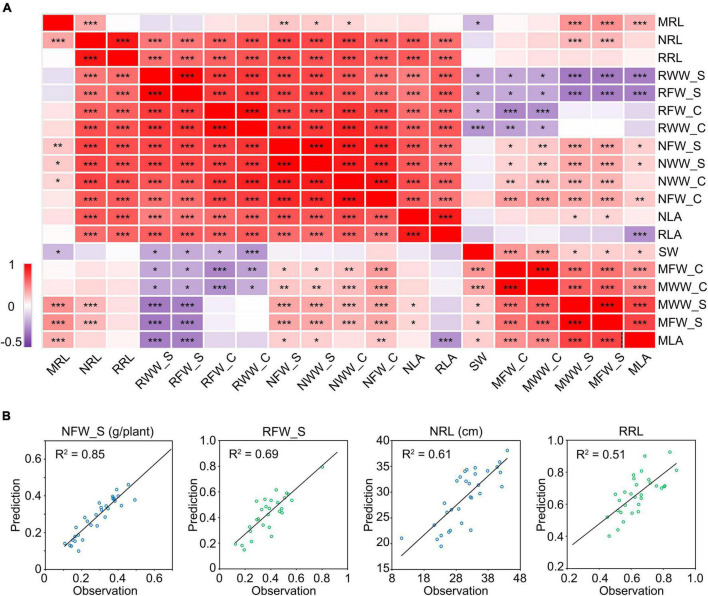
Correlation analysis between salt stress-related traits. **(A)** Correlation matrix between salt stress-related traits. The numbers in the squares indicate the Pearson correlation coefficients. Data were collected from 10 seedlings of 146 different cultivars grown for 10 days under mock or 175 mM NaCl treatment. *P < 0.05; **P < 0.01; ***P < 0.001. **(B)** Regression curve between the predicted value and observed value. The prediction was performed in a new group including 32 cultivars. The first letter “M” in the index name means “mock treatment,” “N” means “NaCl treatment” and “R” means “relative index” (NaCl treatment/mock treatment). The suffix “_C” represents “cotyledon” and “_S” represents “shoot.” RL, root length. LA, leaf area. FW, fresh weight. WW, water weight. SW, seed weight.

To find out the relationship among these phenotypic indices, we performed a correlation analysis. We observed significant positive correlations between the phenotypic indices of plants grown under the same conditions while the correlation coefficients varied (Figure 6A). This implies that the phenotypic indices are related to each other and are different. Notably, the cotyledon-related indices showed very high correlation coefficients with some typical salt tolerance-related indices. For example, the WW of cotyledons under salt stress (NWW_C) showed correlation coefficients of 0.90 and 0.74 with the PRL and FW of shoots under salt stress (NRL and NFW_S), respectively (Figure 6A). It showed similar results with the relative WW of cotyledons (RWW_C). To explore whether the NWW_C or RWW_C can be used to predict the other salt tolerance indices, we constructed the one-dimensional linear regression equations (Supplementary Figure 7) and used them to predict the NFW_S, RFW_S, NRL, and RRL in a new sample group which included 32 cultivars. As a result, the predicted NFW_S showed a coefficient of determination up to 0.85 with its observed value. For RFW_S, NRL, and RRL, the coefficient of determination was 0.69, 0.61, and 0.51, respectively (Figure 6B). These results showed that the moisture in cotyledons could be used to predict the shoot- or root-related indices in soybean salt tolerance screens.

**FIGURE 7:**
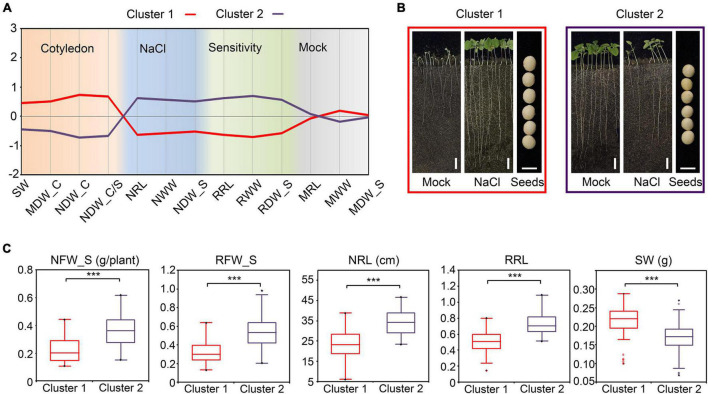
Agglomerative hierarchical clustering of salt stress-related traits in soybean **(A)** The profile plot of the agglomerative hierarchical clustering of salt stress-related traits in soybean. Analysis was performed with the standardized indices. **(B)** Phenotypes of the representative cultivar for Cluster 1 and Cluster 2. Images of the whole seedlings from the soybean cultivars grown in WinRoots for 10 days. Bar, 5 cm in the whole-seedling images. Bar, 5 mm in the seeds images. **(C)** The comparison between the indices of Cluster 1 and Cluster 2. ***P < 0.0001 (*n* = 146), un-paired Student’s *t*-test.

For dry seeds, the seed weight (SW) approximately equals the amount of dry material in cotyledons. In correlation analysis, the SW showed a significant negative correlation with the relative FW in shoots (RFW_S) and the relative WW in shoots (RWW_S) (Figure 6A), which suggested that the dry material in cotyledons also correlate the salt tolerance of plants. We further performed agglomerative hierarchical clustering with the dry-weight-related indices of cotyledons and other salt-tolerance-related indices among the 146 cultivars. As a consequence, the population was grouped into two major clusters (Figure 7A and Supplementary Figure 8). The cultivars in cluster 1 shared common characters such as heavier cotyledons (before germination and after the NaCl or mock treatment), higher salt tolerance, and lower salt sensitivity relative to the cultivars in cluster 2 (Figures 7B,C). As taking off a single cotyledon from a young seedling would not affect its viability, these results demonstrated that the character of cotyledons could serve as a non-destructive indicator for salt tolerance estimation in soybean.

In conclusion, the WinRoots system provides uniform and controllable soil stress conditions for seedling growth, can be used for high-throughput cultivation and phenotyping under soil stress, and helps provide accurate and diverse soil stress-related phenotypic data. WinRoots therefore offers an improved method to analyzing complex traits such as soil stress.

## Discussion

The collection of high-quality data in a high-throughput manner is essential for the study of complex traits. Here, we described WinRoots, a high-throughput crop seedling cultivation and phenotyping system based on a soil matrix, and its application to whole-seedling phenotyping. Capitalizing on the highly controlled and homogeneous soil salt stress environment provided by WinRoots, we collected and analyzed a cultivar of image- and physiological-based traits of soybean seedlings and demonstrated its application potential for high-throughput phenomics studies under soil stress conditions.

### WinRoots System Enables the Accurate Quantification of the Whole-Seedling Phenome

Prerequisites to achieving an accurate quantification of the plant phenome are high-throughput plant cultivation and fast and accurate phenotype collection. In the case of the phenotyping platform GROWSCREEN-Rhizo, 72 individual plants can be grown at once, with a phenotyping rate of 60 plants per hour (Nagel et al., 2012). The high-throughput rice phenotyping facility (HRPF) was reported to have the capacity to hold 5,472 pots, while its associated rice automatic phenotyping platform (RAP) can collect at least 15 agronomic traits at the speed of 80 pots each hour (Yang et al., 2014). The WinRoots system can accommodate 2,160 soybean seedlings (about 12-day-old at normal condition) simultaneously in a 12-m^2^ climate chamber, achieving a growth density of 180 soybean plants per square meter. With the use of an RGB digital camera, the WinRoots system can acquire images for at least 800 seedlings every hour, while the hyperspectral camera can collect 720 canopy images or images from 81 detached leaves *in vitro* per hour with the human factor as the limitation (Figure 1). Thus, the WinRoots system allows (1) high-throughput plant cultivation in soil using only a small footprint and (2) fast acquisition of digital traits from whole seedlings.

Obtaining high-quality phenotypic data is both a prerequisite and a very challenging aspect of performing phenotype-genotype association analyses to identify loci underlying quantitative traits. For rice and cotton (*Gossypium hirsutum*), high-quality phenotypic data obtained with high-throughput phenotyping platforms were subjected to genome-wide association analysis (GWAS), which not only identified the positions of known traditional quantitative traits, but also revealed many new loci, highlighting the strong advantages of this method (Yang et al., 2014; Al-Tamimi et al., 2016; Li B. et al., 2020). Here, the high-throughput multi-phenotypic analysis revealed that cotyledon-related indices as indicators for whole-seedling soybean salt tolerance estimation. We precisely predicted the other salt tolerance-related indices using the cotyledon-related indices (Figure 6). This indicates that the phenotypic indices obtained with the WinRoots system can tease apart the standing variation between soybean cultivars, thereby providing high-quality phenotypic data for later GWAS approaches.

Taken together, the WinRoots system is a powerful tool for the accurate quantification of phenotypes in a high-throughput manner.

### Application of the WinRoots System to Crop Phenomics Studies Under Soil Stress

The development of phenotyping systems that can provide stable and uniform soil stress conditions is one of the main technical challenges in high-throughput crop phenomics under soil stress conditions. Currently, most crop phenotyping studies assessing the effects of soil stress are conducted in the field. However, the uneven distribution of salinity, heavy metal ion concentrations, water content, and other environmental factors in the field severely hinder the evaluation of stress tolerance in crops. It therefore is often necessary to collect phenotypic and environmental data across multiple locations and seasons with high replication, which greatly increases the research cost (Luo et al., 2017; Tardieu et al., 2017; Morton et al., 2019). In crop phenomics studies, several methods have been developed to limit the impact of environmental heterogeneity on plant phenotypes. For example, the Scanalyzer 3D phenotyping platform maintains a constant water moisture level in the soil throughout cultivation with an automated weighing and watering system that adjusts irrigation to compensate for evaporation (Al-Tamimi et al., 2016). The Phenoscope system avoids environmental heterogeneity by rotating pots continuously, resulting in an improved reproducibility for phenotypic data (Tisne et al., 2013). In contrast to these platforms, the WinRoots system uses conductivity monitoring and a liquid circulation system to achieve uniformity of soil stress conditions within and between cultivation units. Soil conductivity under three salt stress conditions was well controlled and maintained as uniform in the WinRoots system (Figure 4A and Supplementary Figure 2). Additionally, the WinRoots system is very flexible in terms of applied stress conditions, as multiple stressors may be imposed on seedlings to meet experimental requirements (for example, we exposed seedlings to four levels of salt stress conditions in this study). Similarly, the number of cultivation units and liquid circulation systems can be configured to accommodate the specifications of the greenhouse and climate chamber, so as to meet the needs of diversified crop soil stress research.

The effects of soil stress on phenotypes are multi-level and multi-faceted. However, due to the lack of powerful phenotypic tools, most past studies on crops experiencing soil stress have been based on a single phenotype (Mano and Takeda, 1997; Campbell et al., 2015; Awlia et al., 2016; Luo et al., 2017). In recent years, with the emergence of high-throughput phenotyping platforms, multi-phenotype studies have offered a number of advantages for elucidating complex traits (Yang et al., 2014; Al-Tamimi et al., 2016; Li B. et al., 2020). For example, Li B. et al. (2020) used an automated phenotyping platform to collect 119 image-based digital traits (i-traits) on natural populations of cotton grown under drought stress conditions and successfully identified 390 drought-related quantitative trait loci (QTLs) by GWAS (Li B. et al., 2020). Our study established that several phenotypic indices were highly correlated in soybean seedlings under salt stress, including root length, fresh weight, and water content, while others were not, such as leaf area and emergence rate. Our results further indicated that phenotypes can be correlated or independent, reflecting the complexity of the phenotypes associated with salt tolerance (Supplementary Figure 9). Therefore, it is critical to collect multiple phenotypes rather than a single one for the study of complex traits. The WinRoots system can collect all phenotypic data based on RGB images, hyperspectral data, and plant materials. For instance, data for root length, plant height, leaf size, and color can be extracted from RGB images; the vegetation index and chlorophyll index can be obtained from hyperspectral data; dry and fresh weight and transcriptomics data can be obtained from the plant materials after this phenotyping. In addition, the WinRoots system can monitor crops in real time with sensors (such as RGB cameras and hyperspectral imaging spectrometers) and record phenotypic changes over time (Figure 4B) to analyze the dynamics of crop phenotypes. In conclusion, the WinRoots system allows the acquisition of multiple types of phenotypic data in a high-throughput manner from whole plants under controlled soil stress conditions and as such is well suited to meet the needs of high-throughput crop phenomics studies under soil stress.

### Perspective and Challenges in Using the WinRoots System

The WinRoots system delivers high-throughput cultivation of crops under controlled soil stress conditions, as well as the instant acquisition of high-throughput and high-quality whole-plant phenotypic data. It will help integrate phenotypes, genotypes, and other omics data sources in a holistic approach to decipher the genetic regulatory networks and mechanisms underlying soil-stress-related traits and hasten precision crop breeding. Compared to previously reported high-throughput crop cultivation and phenotyping systems, the WinRoots system is flexible and offers controllable soil stress settings, while also saving space and cost. It has a wide potential applicability in medium-size crops such as wheat (*Triticum aestivum*), soybean, and rice, as well as large crops such as maize (*Zea mays*), because it can be configured to different sizes and specifications of visual incubators.

The WinRoots system can obtain multiple phenotypic data by simulating soil stress conditions. In the future, it should be possible to integrate WinRoots with field phenotypes to obtain more production-guiding phenotypic indicators and thus speed up phenotypic screening and shorten the breeding cycle. Besides RGB cameras and hyperspectral imaging spectrometers, the WinRoots system can carry more sensors, such as chlorophyll fluorometers and infrared thermal imaging cameras, to expand the types of phenotypes being recorded. Additionally, the development of automatic image-based phenotyping analysis software is necessary to accommodate the volume of data generated, using machine learning methods to improve the efficiency of phenotypic analysis. The final version of this high-throughput and highly efficient phenotyping platform for crop phenomics studies under soil stress will greatly promote the development of plants with resistance to soil stress in crop breeding programs and related fundamental research.

## Data Availability Statement

The original contributions presented in the study are included in the article/Supplementary Material, further inquiries can be directed to the corresponding author/s.

## Author Contributions

YZ performed most of the experiments, data analysis and manuscript writing. WZ performed part of the experiments, data analysis and manuscript writing. QC assisted with the phenotypic data collecting. XZ, JY, TX, WS, XD, LW, JW and FZ performed part of the experiments. FX and XZ assisted with setting up the system. SL designed the system and the experiment, supervised the experiments, data analysis and writing. All authors contributed to the article and approved the submitted version.

## Conflict of Interest

The authors declare that the research was conducted in the absence of any commercial or financial relationships that could be construed as a potential conflict of interest.

## Publisher’s Note

All claims expressed in this article are solely those of the authors and do not necessarily represent those of their affiliated organizations, or those of the publisher, the editors and the reviewers. Any product that may be evaluated in this article, or claim that may be made by its manufacturer, is not guaranteed or endorsed by the publisher.

## Footnotes

1
https://imagej.net/
