# Supplementary material for: WinRoots: A High-Throughput Cultivation and Phenotyping System for Plant Phenomics Studies Under Soil Stress
Source: Front Plant Sci. 2022 Jan 28;12:794020. doi: 10.3389/fpls.2021.794020 (PMC8832124; doi:10.3389/fpls.2021.794020)
Supplement: Supplementary file 1 [file Data_Sheet_1.docx]

**
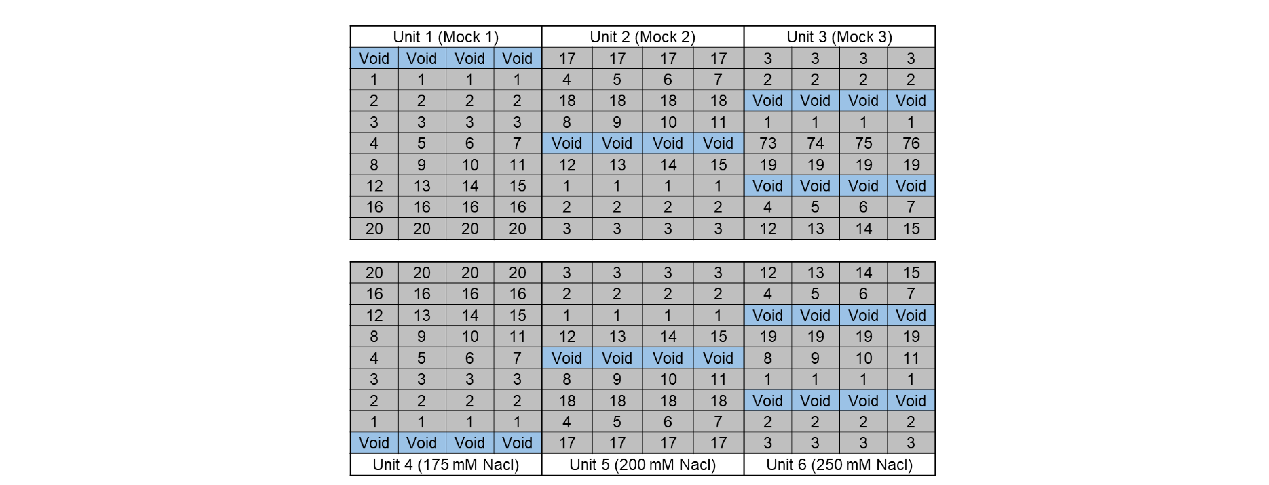
**

**Figure S1. Planting design.**

Each number represents a variety. 'Void' indicates an empty site. Units 1–3, mock treatment; Unit 4, 175 mM NaCl; Unit 5, 200 mM NaCl; Unit 6, 250 mM NaCl.


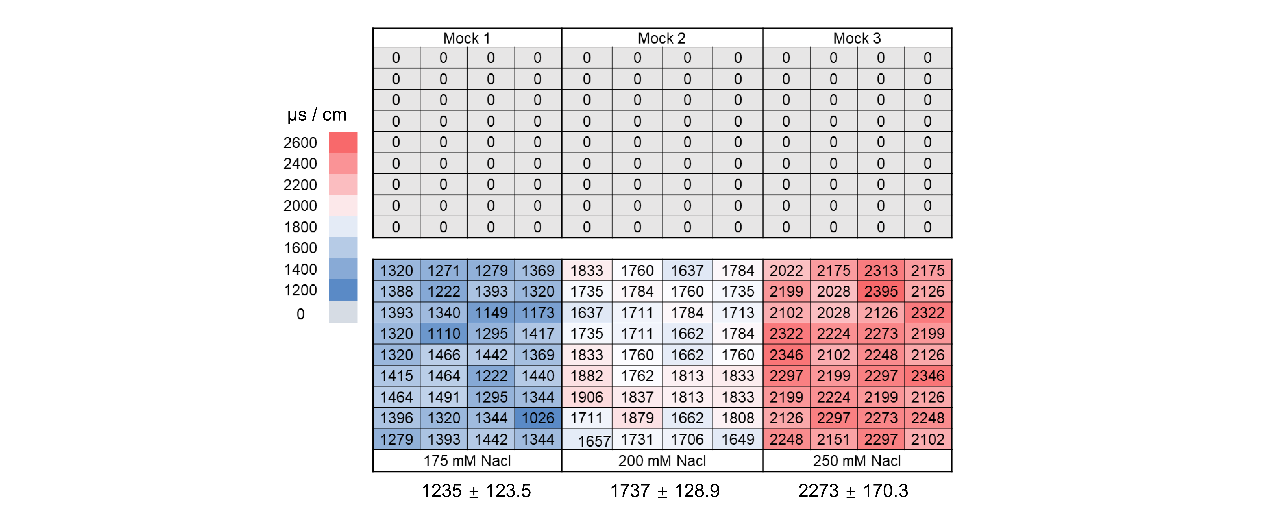


**Figure S2.** **Electrical conductivity map of the soil surface in the WinRoots system (1 day before planting)**

Each number indicates the electrical conductivity at an individual sample site within a culture case. The numbers below the image represent the average electrical conductivity ± SD (*n* = 36).

**
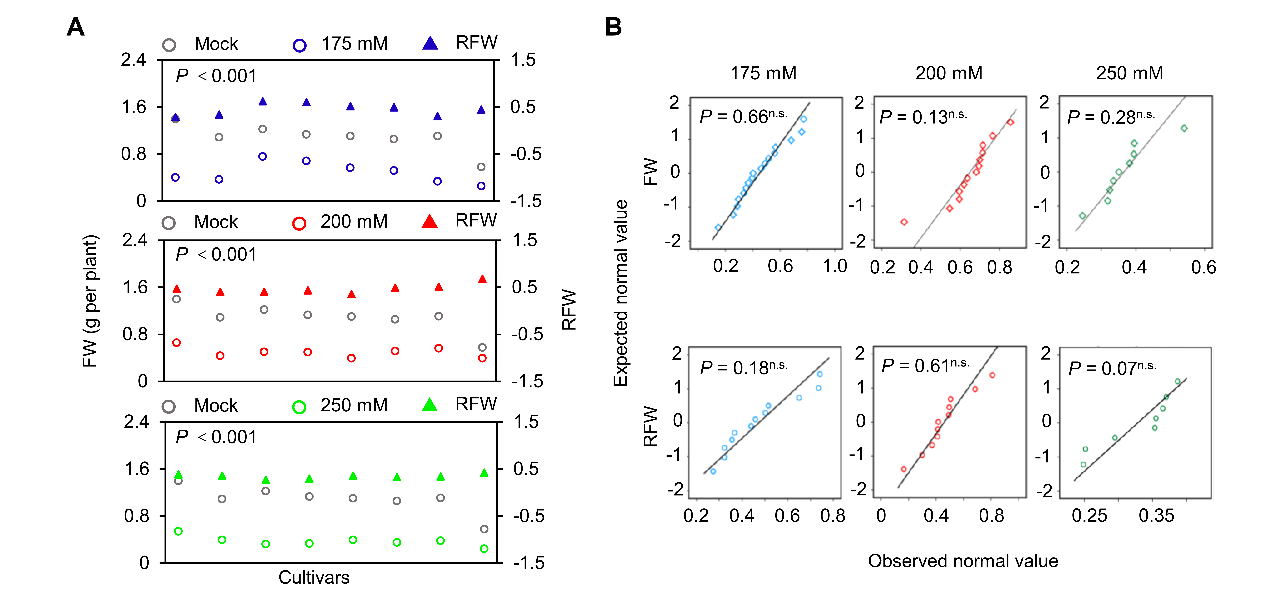
**

**Figure S3. Determination of fresh weight.**

(**A**) Scatter distribution map of the fresh weight (FW, circles) and relative fresh weight (RFW, triangles) of soybean seedlings. Grey, blue, red, and green represent the soybean seedlings growing under mock treatment or NaCl treatment of 175, 200, or 250 mM, respectively. Each circle represents the average value (*n* = 8) of FW of a cultivars. Each triangle represents the value of the RFW of a soybean cultivar. Data were collected 10 days after planting. A paired Student's *t*-test was conducted between the mock and NaCl-treated seedlings to test for significance. (**B**) Quantile-quantile plots of FW and RFW. Normality of the data was tested using the Shapiro-Wilk test. *P*, *P* value; n.s., not significant. The planting design is shown in Figure S1.


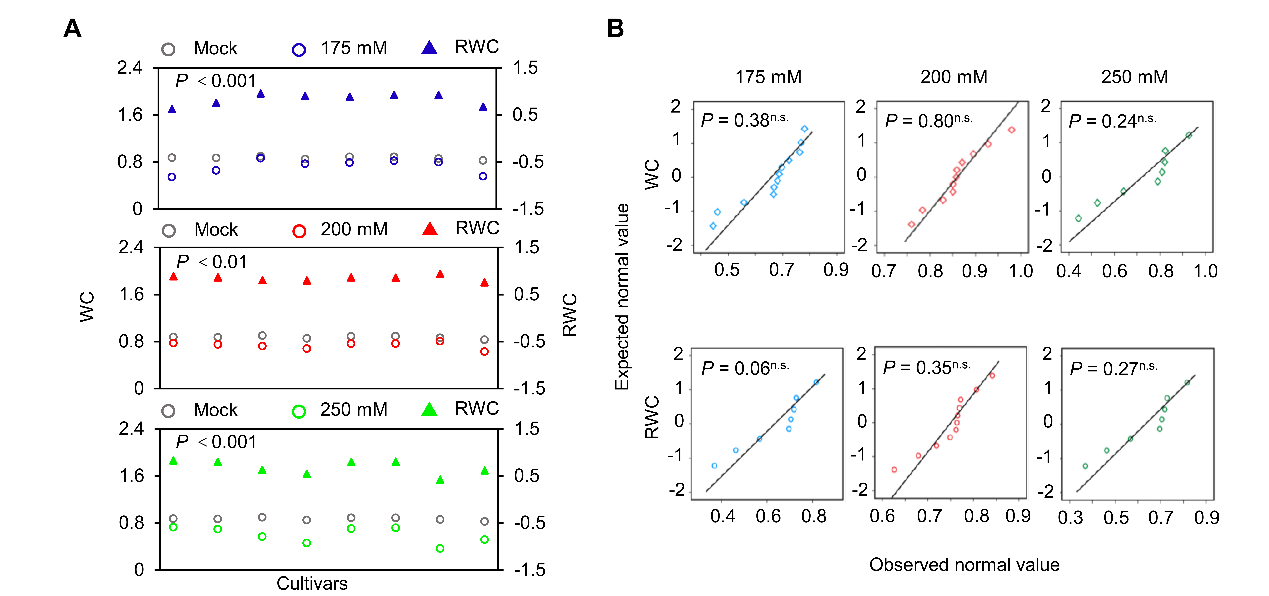


**Figure S4. Determination of water content.**

(**A**) Scatter distribution map of the water content (WC, circles) and relative water content (RWC, triangles) of soybean seedlings. Grey, blue, red, and green represent the soybean seedlings growing under mock treatment or NaCl treatment of 175, 200, or 250 mM, respectively. Each circle represents the average value (*n* = 8) of WC of a cultivars. Each triangle represents the value of the RWC of a soybean cultivar. A paired Student's *t*-test was conducted between the mock and NaCl-treated seedlings to test for significance. (**B**) Quantile-quantile plots of WC and RWC. Normality of the data was tested using the Shapiro-Wilk test. *P*, *P* value; n.s., not significant. The planting design is shown in Figure S1.

**
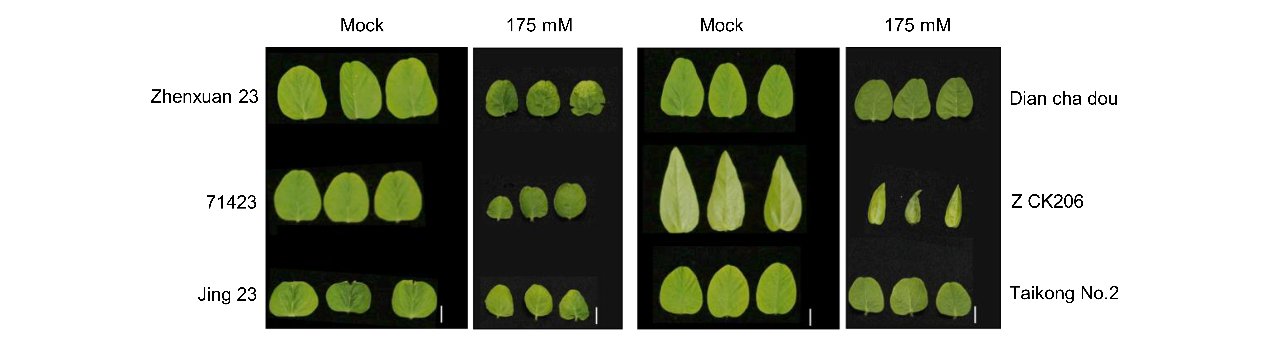
**

**Figure S5. Representative images of detached leaves.**

Leaves were collected from seedlings 9 days after growth under mock or 175 mM NaCl treatment. Shown are unifoliolate leaves from three independent seedlings per cultivar and growth condition. Bar = 1 cm.


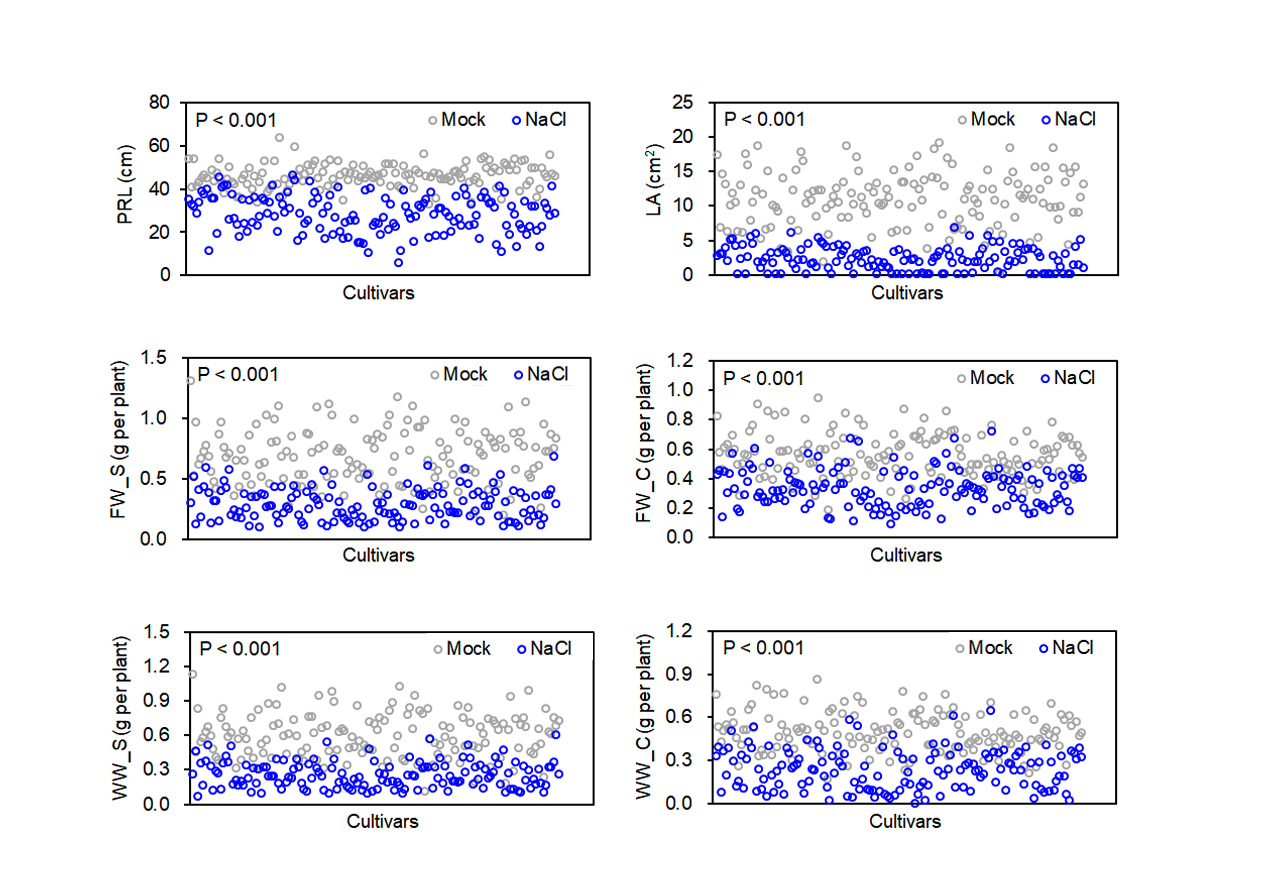


**Figure S6. Scatter distribution map of three physiological traits collected on soybean seedlings.**

Grey and blue dots represent the traits under mock or 175 mM NaCl growth conditions, respectively. Data were collected 10 days after planting. Each dot represents the average value (*n* = 8) of the relevant trait for 146 different cultivars under corresponding conditions. A paired Student's *t*-test was conducted between the mock and NaCl-treated seedlings to test for significance. *P*, *P* value*.*


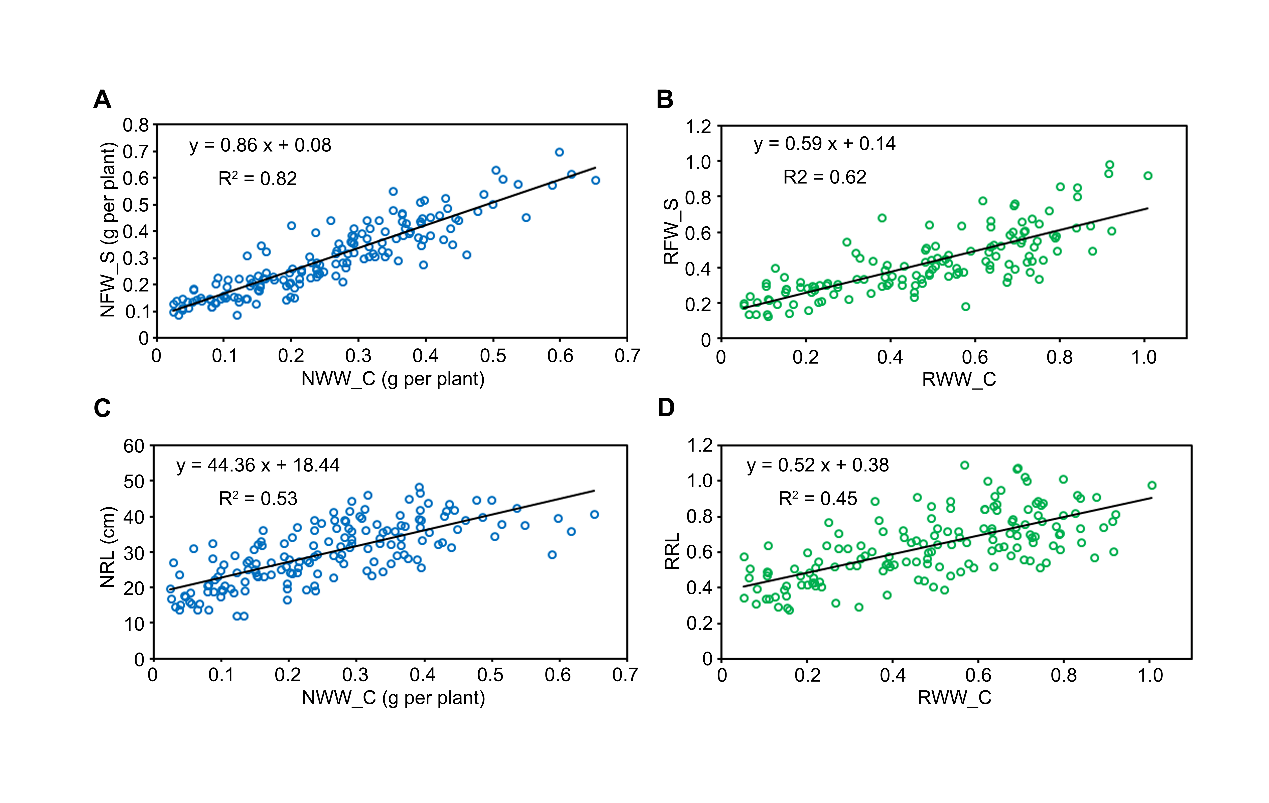


**Figure S7. The linear regression equations.**

The linear regression equations were constructed between NWW_C and NFW_S (**A**), RWW_C and RFW_S (**B**), NWW_C and NRL (**C**), and RWW_C and RRL (**D**), respectively. The data were obtained from the 146 soybean cultivar.


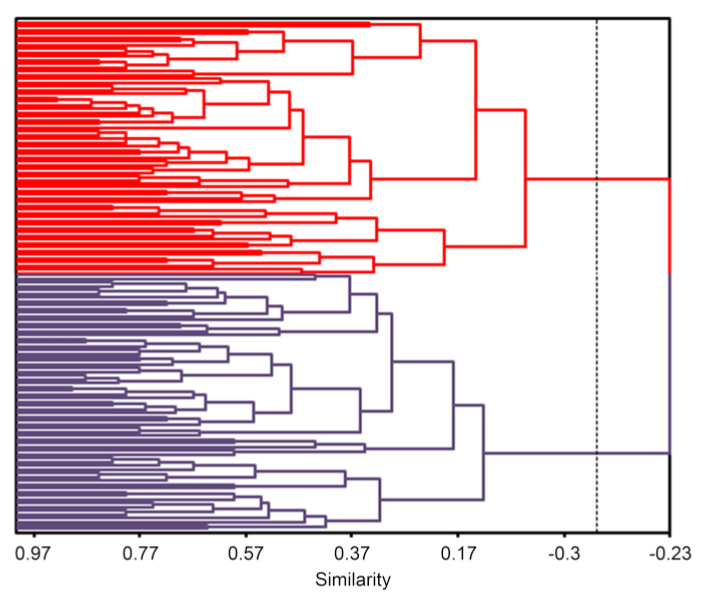


**Figure S8. Clustering dendrogram.**

The clustering dendrogram was constructed based on the phenotypic similarity of the 146 soybean cultivars.


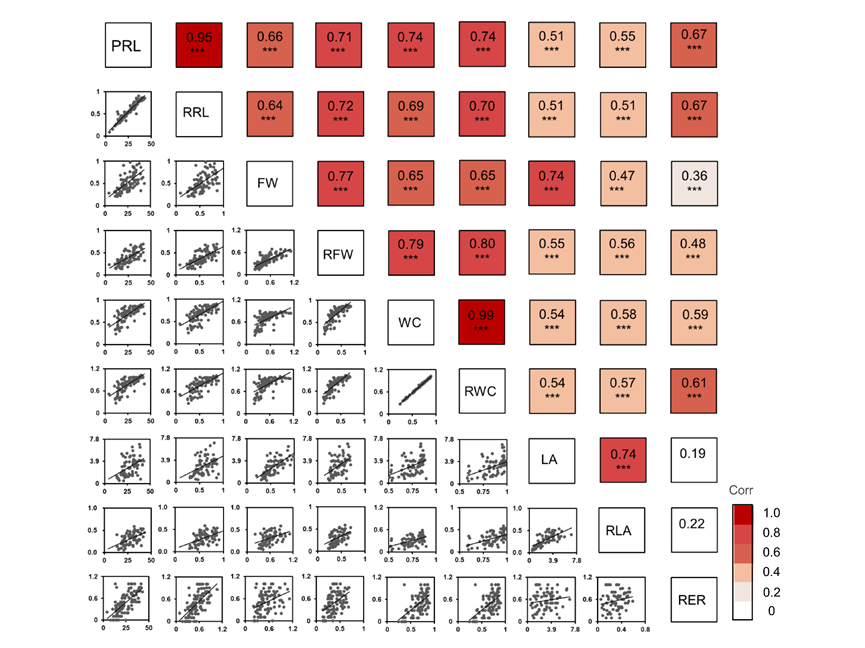


**Figure S9. Correlation matrix between salt stress-related traits.**

The numbers in the squares indicate the Pearson correlation coefficients (Corr). Data were collected from 10 seedlings of 108 different cultivars grown for 10 days under mock or 175 mM NaCl treatment. **P* ＜ 0.05; ***P* ＜ 0.01; ****P* ＜ 0.001. PRL, primary root length; RRL, relative primary root length; FW, fresh weight; RFW, relative fresh weight; WC, water content; RWC, relative water content; RLA, relative leaf area; RER, relative emergence rate.
